# The efficacy of two different oral hygiene regimens on the incidence and severity of oral mucositis in pediatric patients receiving hematopoietic stem cell transplantation: A prospective interventional study

**DOI:** 10.1111/scd.12525

**Published:** 2020-09-21

**Authors:** Sarah Mubaraki, Sharat Chandra Pani, Amal Alseraihy, Hassan Abed, Zikra Alkhayal

**Affiliations:** ^1^ Department of Preventive Dentistry Riyadh Colleges of Dentistry and Pharmacy Riyadh Saudi Arabia; ^2^ Department of Pediatric Dentistry and Orthodontics The University of Western Ontario London Canada; ^3^ Department of Pediatric Hematology∖Oncology King Faisal Specialist Hospital and Research Center Riyadh Saudi Arabia; ^4^ Department of Sedation and Special Care Dentistry Guy's & St Thomas’ Foundation Trust London UK; ^5^ Faculty of Dentistry Department of Basic and Clinical Oral Sciences Umm Al‐Qura University Makkah Saudi Arabia; ^6^ Department of Dentistry King Faisal Specialist Hospital and Research Center Riyadh Saudi Arabia

**Keywords:** chemotherapy, oral hygiene, oral mucositis, stem cell transplantation

## Abstract

**Aims:**

This prospective interventional study aimed to assess the efficacy of supersaturated calcium phosphate rinse and the use of an extra‐soft toothbrush twice a day when added to the existing oral hygiene protocol regimen (0.12% chlorhexidine gluconate + 3% sodium bicarbonate + nystatin 5000 U/mL) in reducing the severity of oral mucositis among pediatric patients receiving chemotherapy for the hematopoietic stem cell transplant.

**Methods:**

Forty‐five patients that received chemotherapy for the hematopoietic stem cell transplant were randomly allocated to three groups of 15 patients each. Group A was advised to follow the existing oral hygiene protocol regimen (Control), group B was advised to brush their teeth twice daily using an extra‐soft toothbrush and to follow the control regimen, and lastly group C was advised to use supersaturated calcium phosphate rinse and to follow the control regimen. Oral mucositis was recorded according to World Health Organization criteria from the day of admission (day 1) to the day of discharge (day 28). The incidence of oral mucositis between the three groups was compared using the Kruskall‐Wallis test while the severity of oral mucositis between the three groups was compared using a one‐way ANOVA test.

**Results:**

The results of the study showed no significant difference in the incidence of oral mucositis between the three groups; however, there was a lower severity of oral mucositis in the supersaturated calcium phosphate rinse group when compared to the control group or the group who used an extra‐soft toothbrush with the control regimen.

**Conclusion:**

Although marginally fewer cases and lower severity of oral mucositis was observed in the group using supersaturated calcium phosphate rinse, the lack of statistical significance suggests that the evidence for their use is not conclusive. The results of this study also showed that the introduction of an extra‐soft toothbrush into the oral hygiene regimen did not significantly reduce the incidence of oral mucositis and may actually be responsible for an increase in the severity of oral mucositis.

## 1 INTRODUCTION

Hematopoietic stem cell transplant (HSCT) is defined as the infusion of multipotent stem cells derived from bone marrow, cord blood or peripheral blood to reconstitute the hematopoietic system.^1^ HSCT is used to treat several hematological disorders.^2^ Oral mucositis (OM) is one of the side‐effects developed post‐HSCT.^1^ It is defined as an inflammation of oropharynx resulting from cancer therapy typically manifesting as atrophy, swelling, erythema and ulceration.^1^ It affects 80% of children undergoing HSCT,^3^ and usually occurs between two and 18 days following the initiation of the chemotherapy.^2^ It is generally accepted that the pathophysiology of OM is a complex multistage phenomenon that can result from damage to the oral mucosal cells by free radicals produced by the drugs used in chemotherapy.^4^ OM is a painful and debilitating adverse effect, which can cause a significant reduction in the affected patient's quality of life (QoL), increase in hospital stay and other complications such as malnutrition in children due to poor oral intake.^5^ Thus, studies related to protocols to lessen these effects are necessary.

The management described in the literature in the prevention and treatment of OM has focused extensively on the symptomatic relief through topical application of anesthetics, obtundents, antifungals, and even placebo combinations.^6^ There has been a tendency to accept a combination of chlorhexidine mouth‐rinse, a bis‐guanidine antimicrobial, with the addition of antifungal such as nystatin as a standard of oral care in many hospitals.^7^ There is also some clear indication that maintenance of good oral hygiene may help alleviate the symptoms of OM.^8^ Oral care improvement has a significant effect in the reduction of developing moderate/severe OM without causing an increase in septicemia and infection in the oral cavity.^9^ Moreover, the relationship between dental plaque accumulation and OM has been also investigated and the results showed that dental plaque was a known causative factor in oral inflammation and determinant of OM incidence.^9^

While there is no argument in the literature about the need for oral hygiene, there is considerable argument about how best to achieve it. It is a well‐documented fact that physical methods such as toothbrushes are far more effective than chemical methods alone.^10^ The American Academy of Pediatric Dentistry recommended that patients should maintain good oral hygiene by brushing their teeth and tongue two to three times daily, regardless of their hematological status.^11^ Yamagata et al^12^ recommended patients undergoing chemotherapy with or without HCST to brush their teeth to prevent gingival inflammation and bacterial colonization. Children with poor oral hygiene can use alcohol‐free chlorhexidine gluconate until gingival health is improved or OM develops. It has also been reported that if a patient develops moderate to severe OM and cannot tolerate the soft nylon toothbrush, then sponge brushes soaked in chlorhexidine can be used until regular toothbrushing can resume.^2^

SCPR were introduced in 2009 and they have been shown to reduce the symptoms of OM in patients receiving HSCT by lubricating the oral cavity, which can minimize severe OM.^13^ SCPR is a natural electrolyte solution containing calcium and phosphate ions that resemble the ionic and pH balance of saliva.^13^ Theoretically, the highly concentrated calcium and phosphate ions diffuse into intercellular spaces of the oral epithelium. The calcium ions play a major role in the inflammatory process, the blood‐clotting cascade, fibrin production, and tissue repair. Phosphate ions are important in facilitating intracellular signaling and regulating the voltage potential inside the cell; both mechanisms are important in repairing and protecting damaged mucosal surfaces.^14^

This prospective interventional study aimed to assess the efficacy of SCPR and the use of an extra‐soft toothbrush (ESTB) twice a day when added to the existing oral hygiene protocol regimen (0.12% chlorhexidine gluconate + 3% sodium bicarbonate + nystatin 5000 U/mL) in reducing the severity of OM among pediatric patients receiving chemotherapy for HSCT.

## 2 MATERIALS AND METHODS

This prospective interventional study was conducted from December 2014 to December 2015 on children who received HSCT at the Hematology‐Oncology and Stem Cell Transplantation Department at King Faisal Specialist Hospital and Research Center (KFSHRC). This center is the main one for HSCT in Riyadh, Saudi Arabia. This study was approved by the Ethics Committee of Riyadh Elm University and King Faisal Specialist Hospital and Research Center. A written informed consent was obtained from each parent/guardian and the procedure was explained to the child. All data collected was part of the routine medical practice for managing patients receiving HSCT at the KFSHRC. The trial was registered on the clinical trials registry of the National Institutes of Health (NIH) (registry number: NCT02662374).

### 2.1 Study design and patient selection

This was a prospective interventional study with a parallel design conducted. The patients were selected using a random distribution into either the control or one of the two intervention groups. Patients were matched for diagnosis and age.

### 2.2 Inclusion and exclusion criteria

The inclusion criteria were patients aged seven to 10 years old who were receiving allogeneic transplants from a completely matched donor, and patients who were receiving a combination of three or more of the following agents; fludarabine, busulphan, cyclophosphamide, methotrexate, cyclosporine, or antithymocyte globulin. Patients who were receiving autologous cord blood stem cell transplants or radiation, and patients who had received previous graft or organ transplants were excluded from the study.

### 2.3 Procedure

The existing oral hygiene protocol for all patients receiving HSCT at the center of the study comprised a combination of 0.12% chlorhexidine gluconate (Clorasept, Spiaco Addwaeih, Riyadh, KSA) and sodium bicarbonate (3% aqueous solution USP) administered as a mouthwash four times daily concomitantly with 5000 IU of nystatin swished and swallowed by the patient. Patients were allowed to rinse with sterile water whenever they requested. Patients receiving this protocol comprised the control group (Group A). Group B comprised patients who were advised to use an extra‐soft toothbrush (Slim Soft 0.01 mm bristles 2016 Colgate‐Palmolive New York, NY, USA) and water twice a day with close supervision from the PI in addition to the above mentioned control protocol. Group C comprised patients who were instructed to use a small amount of supersaturated calcium oral spray (MoiStir, Kingswood Labratories, Inc, Indianapolis, IN, USA) and to swish for thirty seconds then spit out four times a day in addition to the existing control oral hygiene protocol. The principal investigator, who is an experienced pediatric dentist, instructed all the study groups and their parents on the oral hygiene protocols.

### 2.4 Recording of data

The patient's age, gender, type of disease, and types of chemotherapeutic agents used for the treatment were collected from the patient's hospital records and transferred to a customized data sheet using Microsoft Excel (Microsoft Corp, San Jose CA, USA). Incidence of OM was calculated based on the presence or absence of OM. Severity of OM was calculated using the WHO scale.^15^ The severity of OM on each day was cumulated to arrive at a composite severity score. Each patient had a unique number, thus there were no data identifying the patients on the data sheets. All children in the study had no OM and a score of 0 using the WHO scale. The recording of the scale was performed by two investigators (SA and ZA) at different times with interrater reliability of 80%. It should also be noted that the grading of OM is performed by the oncology team as part of routine care. Patients’ tolerance of the oral hygiene was defined as the ability of the patient to complete the described protocol for all of the days the child spent in the hospital. Patients' lack of assent to administer the oral hygiene protocol was defined as a lack of tolerance.

### 2.5 Statistical analyses

Chi square test was used to determine significance between the three groups. The Kruskul‐Wallis was used to determine the equal distribution of the chemotherapeutic regimen received. The significance difference in the incidence of OM was calculated using the Kruskul‐Wallis test. The significance in the severity of the OM was calculated using the one‐way ANOVA test. The patient tolerance of the different regimen before and after chemotherapy was tabulated. The significance of difference in tolerance of the regimen before and after chemotherapy was measured using the Wilcoxon Sign Rank Test. All analyses were performed using SPSS version 21 data processing software (IBM Corp., Armonk, NY, USA).

### 2.6 Sample size calculation

The sample size calculation was done using the G:Power sample power calculator (version.3.0, Kiel, Germany). It was observed that the post hoc power achieved for 45 patients using the bivariate tests and an effect size of 0.5 (moderate effect size) was 0.92.

## 3 RESULTS

One‐hundred and forty‐six patients were assessed for eligibility. Of these, 101 patients were excluded as they did not meet the inclusion criteria, while 45 were eligible to be included in the study. The sample comprised 20 males and 25 females. The mean age of the population was 7.7 years old (*SD* = 3.12). The females, mean age = 8.3 years old (*SD* = 2.90), were slightly older than the male mean age = 8.1 years old (*SD* = 3.31). However, these differences were not statistically significant (*t* = –1.208, *P* = 0.234).

Table 1 presents the characteristics of the sample according to group and type of hematological disorders. The patients were evenly distributed across the different groups according to their primary diagnosis. It was also observed that the patients across all groups received a similar chemotherapeutic regimen and immunosuppressive drugs with no significant difference in the distribution of chemotherapeutic regimen among groups (Table 2).

**TABLE 1:** Characteristics of the sample according to group and type of hematological disorder

	Control (*N* = 15)	ESTB (*N* = 15)	SCPR (*N* = 15)	Total (*N* = 45)
Type of hematological disorder	*N* (%)	*N* (%)	*N* (%)	*N* (%)
Aplastic anemia	2 (13.3%)	2 (13.3%)	2 (13.3%)	6 (13.3%)
Acute Lymphoblastic leukemia	2 (13.3%)	2 (13.3%)	2 (13.3%)	6 (13.3%)
Acute myeloblastic leukemia	2 (13.4%)	3 (20.0%)	2 (13.4%)	7 (15.6%)
Fanconi anemia	3 (20.0%)	2 (13.4%)	3 (20.0%)	8 (17.8%)
Sickle cell disease	3 (20.0%)	3 (20.0%)	3 (20.0%)	9 (20.0%)
Thalassemia	3 (20.0%)	3 (20.0%)	3 (20.0%)	9 (20.0%)

ESTB, extra‐soft tooth brush; SCPR, supersaturated calcium phosphate rinse.

**TABLE 2:** Types of chemotherapeutic agents administered to participants (total = 45)

	Control (*N* = 15)	ESTB (*N* = 15)	SCPR (*N* = 15)	Statistical analysis	
Drug	*N* (%)	*N* (%)	*N* (%)	Chi‐squared test	*P value*
Busulfan	8 (53.3%)	10 (66.6%)	11 (73.3%)	1.328	0.515
Fludarabine	7 (46.7%)	6 (40.0%)	8 (53.3%)	0.524	0.770
Cytosine	15 (100.0%)	12 (80.0%)	11 (73.3%)	4.301	0.116
Antithymocyte globulin	10 (66.7%)	5 (33.3%)	10 (66.6%)	5.065	0.079
Cyclophosphamide	1 (6.7%)	1 (6.7%)	1 (6.7%)	0.000	1.000
Methotrexate	12 (80.0%)	11 (73.3%)	11 (73.0%)	3.859	0.145

ESTB, extra‐soft tooth brush; SCPR, supersaturated calcium phosphate rinse.

Table 3 shows the incidence of OM among participants. The incidence of OM in the population was calculated on the basis of whether a patient developed mucositis or not. When the incidence of OM among the three groups was compared, it was observed that the control group had the greatest incidence of OM while the SCPR groups showed the lowest incidence. However, these differences were not statistically significant.

**TABLE 3:** The incidence of oral mucositis among participants (total = 45)

	Control (*N* = 15)	ESTB (*N* = 15)	SCPR (*N* = 15)	Statistical analysis	
	*N* (%)	*N* (%)	*N* (%)	Chi‐squared test	*P* value
Absent	3 (20.0%)	4 (26.7%)	5 (33.3%)	0.682	0.711
Present	12 (80.0%)	11 (73.3%)	10 (66.7%)		

ESTB, extra‐soft tooth brush; SCPR, supersaturated calcium phosphate rinse.

Comparison of the severity of OM among the different groups showed that the SCPR group showed the lowest severity of OM, followed by the control group. The ESTB group showed the highest severity of OM. However, these differences were not statistically significant (Table 4).

**TABLE 4:** Severity of oral mucositis among participants (total = 45):

	Statistical analysis				
Group	*N* (%)	Mean	*SD*	*F* ::	*P* value
Control (*N* = 15)	12 (80.0%)	5.2	2.66	0.347	0.710
Extra‐soft tooth brush (*N* = 15)	11 (73.3%)	5.9	3.45		
Supersaturated calcium phosphate rinse (*N* = 15)	10 (66.7%)	4.7	4.00		

: Severity calculated by multiplying the grade of mucositis with the number of days observed.

:: One‐way ANOVA.

Table 5 presents participants’ tolerance of the different components of the control regimen before and after HSCT. The significance of difference in tolerance of the regimen before and after was measured using the Wilcoxon Sign Rank Test. The results indicated that there were no significant changes in compliance for the nystatin or sodium bicarbonate. However, there was a significant reduction in the tolerance of chlorhexidine after chemotherapy. When the tolerance to chlorhexidine was observed for each group specifically, it was observed that the tolerance of chlorhexidine was significantly less in the SPCR group (Table 6). When the patient tolerance of the two interventions was compared using the Mann‐Whitney U test, it was observed that post‐chemotherapy, the ESTB group was significantly better tolerated than SCPR (Table 7).

**TABLE 5:** Tolerance of the different components of the control regimen (*N* = 15) before and after the hematopoietic stem cell transplant (HSCT)

		*N*	Mean rank	*P* value
Compliance with chlorhexidine (CHX)	Negative ranks	8^a^	5.00	0.020:
	Positive ranks	1^b^	5.00	
	Ties	36^c^		
	Total	45		
Compliance with sodium bicarbonate (SB)	Negative ranks	6^d^	5.50	0.527
	Positive ranks	4^e^	5.50	
	Ties	35^f^		
	Total	45		
Compliance with + nystatin (NY)	Negative ranks	8^g^	6.00	0.132
	Positive ranks	3^h^	6.00	
	Ties	34^i^		
	Total	45		

^a^ Compliance with CHX – after HSCT < compliance with CHX – before HSCT.

^b^ Compliance with CHX – after chemo > compliance with CHX – before HSCT.

^c^ Compliance with CHX – after chemo = compliance with CHX – before HSCT.

^d^ Compliance with SB – after HSCT < compliance with SB – before HSCT.

^e^ Compliance with SB – after HSCT > compliance with SB – before HSCT.

^f^ Compliance with SB – after HSCT = compliance with SB – before HSCT.

^g^ Compliance with NY – after HSCT < compliance with NY – before HSCT.

^h^ Compliance with NY – after HSCT > compliance with NY – before HSCT.

^i^ Compliance with NY – after HSCT = compliance with NY – before HSCT.

: *P* < 0.05.

**TABLE 6:** Comparison of the tolerance of chlorhexidine (CHX) before and after the hematopoietic stem cell transplant (HSCT)

Group		*N*	Mean rank	Sum of ranks
Control (*n* = 15)	Negative ranks	3^a^	2.00	0.083
	Positive ranks	0^b^	0.00	
	Ties	12^c^		
	Total	15		
Extra‐soft tooth brush (*N* = 15)	Negative ranks	0^a^	0.00	0.317
	Positive ranks	1^b^	1.00	
	Ties	14^c^		
	Total	15		
Supersaturated calcium phosphate rinse (*N* = 15)	Negative ranks	5^a^	3.00	0.025:
	Positive ranks	0^b^	0.00	
	Ties	10^c^		
	Total	15		

^a^ Compliance with CHX – after HSCT < compliance with CHX – before HSCT.

^b^ Compliance with CHX – after HSCT > compliance with CHX – before HSCT.

^c^ Compliance with CHX – after HSCT = compliance with CHX – before HSCT.

: *P* < 0.05.

**TABLE 7:** Comparison of the tolerance of intervention before and after the hematopoietic stem cell transplant (HSCT)

Tolerance of intervention		ESTB	SPCR	*P* value
Before HSCT	<25% compliance	0	0	
				0.539
	25‐50% compliance	0	0	
	50‐75% compliance	1	3	
	>75% compliance	14	11	
After HSCT	<25% compliance	0	0	
				0.011:::
	25‐50% compliance	0	0	
	50‐75% compliance	4	6	
	>75% compliance	11	9	

ESTB, extra‐soft tooth brush; SCPR, supersaturated calcium phosphate rinse.

::: *P* < 0.05.

## 4 DISCUSSION

OM is a common complication in children who are undergoing HSCT. It impacts on their QoL negatively.^5^ As a result, there has been a need to investigate different regimens and protocols to reduce the prevalence and severity of OM in children undergoing HSCT.

It is important to mention that the combination regimen used by the control protocol (ie, chlorhexidine, sodium bicarbonate and nystatin) has been documented to be one of the many standard protocols developed for patients undergoing chemotherapy.^16^ The protocol was used in our center with perceived good results for a long time. The components—sodium bicarbonate, chlorhexidine and nystatin—are implemented against OM when it was formerly regarded as having a bacterial or fungal cause, and are supported by research with small groups and often without controls. The group was certainly considered to be appropriate as control with no specific contraindication. Although no guideline was published in the success of chlorhexidine mouthwash for prevention of OM in children, it is well documented that the use of chlorhexidine is successful in treatment of gingivitis and control of plaque found in children with poor oral hygiene.^16^

Tolerance of an oral hygiene protocol, in this study, was defined as the ability of the child patient to complete the described protocol for all the days. The poor cooperation of children, in addition to low tolerance for mouth‐rinses, illustrated in this study, with using the chlorhexidine or SCPR mouth‐rinse after starting chemotherapy may play a role in previous investigations that were not able to establish a practical guideline in using mouth‐rinses such as chlorhexidine.

The use of an ESTB prescribed in the first intervention group of this study was based on a previously established toothbrushing protocol for medically compromised children.^6^ It has been reported that toothbrushing carries with it the risk of oral mucosal ulceration due to trauma, which may be accompanied by uncontrolled bleeding in hematological compromised children.^6^ Many authorities such as the American Academy of Pediatric Dentistry recommended in their guidelines the use of a soft nylon toothbrush two to three times daily, as intensive oral care is of paramount importance in reducing the risk of developing moderate to severe OM without risk during hematological changes.^11^ Interestingly, the guideline further promotes the use of alcohol‐free chlorhexidine as a method to improve oral gingival health if mechanical methods were not used prior to chemotherapy or in times when a soft toothbrush cannot be tolerated during moderate to severe OM, with the objective of continuing with the antiseptic oral hygiene until toothbrushing can be resumed. Further, in this study the children were being monitored on a daily basis and any possible risk of uncontrolled bleeding or infection secondary to trauma was negligible and was negated by the potential benefits of the toothbrushing. However, the results of this study may indicate that the introduction of an ESTB into the oral hygiene regimen did not significantly reduce the incidence of OM. The increased severity of OM observed in this group warrants further investigation into the role intolerance and/or inability to maintain oral hygiene plays in OM.

SCPR in the second intervention group of our study was marginally effective in reducing the incidence and severity of OM in children undergoing HSCT. Papas et al^17^ found that SCPR is an effective tool in the reduction of OM. Our findings are also similar to those of Ambard et al,^18^ who found no significant difference in the severity of OM between patients on SCPR and those receiving a control rinse.

An interesting observation in this study is the large standard deviation in the intensity of OM across all groups, with the SCPR group showing the highest intra‐group variation and range. Patient compliance with an oral hygiene regimen is critical to the success of such a regimen in hematologically compromised patients.^6^ It was observed that the ESTB and sterile water was significantly better tolerated than the SCPR in patients, especially in days after the bone marrow transplant. This finding was observed in spite of the fact that patients in the SCPR group had a slightly lower incidence and severity of OM. On the other hand, SCPR rinses have been reported by some studies to be well tolerated by patients suffering from OM after HSCT.^6^ Chemotherapy causes nausea, changes in taste, and noncompliance with the established protocols. Although all efforts were directed toward ensuring standardization of the daily amount of mouthwash utilized, there exists inherent variation in the exact correct amount of rinse utilized daily during the study. This, coupled with the decrease in tolerance level for the rinses after chemotherapy, may suggest that in children when using mouthwashes, supervised sponge soaked with the rinse provides an alternative and more effective way in delivering the intended mouth‐rinse.

In this study, although marginally fewer cases and lower severity of OM was observed in the group using SCPR rinses, the lack of statistical significance suggests that the evidence for their use is not conclusive. The results of this study also showed that the introduction of an ESTB into the oral hygiene regimen did not significantly reduce the incidence of OM and may actually be responsible for an increase in the severity of OM. However, this must be presented with caution, given the small sample size and lack of statistical significance. It is also crucial to mention that conducting studies on the prevention of OM for children undergoing HSCT is difficult due to problems with randomization, age of patients, and sampling technique.^19^

## 5 CONCLUSIONS AND RECOMMENDATIONS

The interventional use of ESTB and SCPR as oral hygiene regimens in this prospective study did not have a significant effect on the incidence and severity of OM in pediatric patients receiving HSCT. The results warrant further investigation in a larger controlled trial population and multicenter studies are needed to assess different oral hygiene protocols and their effect in reducing the incidence and severity of OM and hence the QoL in children undergoing HSCT.

## CONFLICTS OF INTEREST

The authors declared that there is no conflict of interest.

## ETHICAL APPROVAL

Ethical approval for the project was obtained from the research center of the King Faisal Specialist Hospital and Research Center, Riyadh, Saudi Arabia (RAC number: 2151139) and from the research center of the Riyadh Colleges of Dentistry and Pharmacy, Riyadh, Saudi Arabia (FPGRP∖43435003∖100).
